# SapTrap Builder: a desktop utility for CRISPR edit design

**DOI:** 10.17912/m4qq-2x02

**Published:** 2018-07-29

**Authors:** Matthew Schwartz, Erik Jorgensen

**Affiliations:** 1 Department of Biology and Howard Hughes Medical Institute, University of Utah, Salt Lake City, Utah 84112-0840

**Figure 1.**
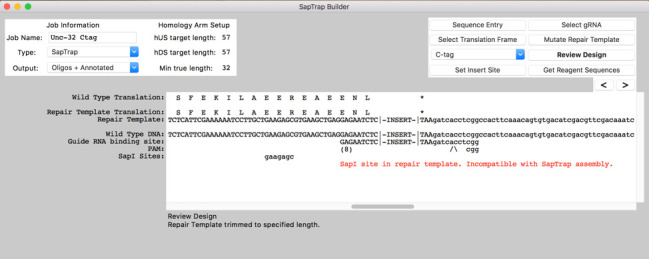
The SapTrap Builder software interface, shown designing a C-terminal tag insertion in *unc-32*.

## Description

Designing and building DNA reagents for CRISPR edits can be complicated as well as tedious. To streamline reagent design for our SapTrap CRISPR plasmid assembly system, we developed the SapTrap Builder software utility. SapTrap Builder provides an intuitive graphical user interface for visualizing the design of reagents for CRISPR-based genetic tag insertion and point mutagenesis. Our program guides users through several steps of CRISPR edit design, alerts the user when incompatibilities arise, and generates sequences of DNA oligomers required for the desired edit. Although primarily developed for use with the SapTrap system, the program can be used to design CRISPR reagents for any protocol.

Using CRISPR/Cas9 to generate random deletions in a gene is quite simple and efficient. CRISPR/Cas9 can also be used to generate specific amino acid changes in a protein or insert genetically-encoded fluorescent tags, but such custom edits require reagents that are complicated to properly design and build. To streamline reagent production, we previously developed SapTrap, which modularizes the design and assembly of targeting vectors for genetic tag insertion in *C. elegans* (Schwartz and Jorgensen, 2016). Now, we introduce companion software, called SapTrap Builder, that guides users through the design of site-specific reagents for the SapTrap system. Using our program, researchers can design reagents for SapTrap targeting vector assemblies in less than one minute for most sites. The program can also be used to select insertion sites and guide RNAs for non-SapTrap strategies.

Designing a specific CRISPR-based edit requires (1) picking a guide RNA, (2) selecting homology arms flanking the insert or edit site, (3) introducing mutations within the homology arms (silent changes if needed to immunize the repair template against further cleavage by Cas9, and if desired, coding changes to the ORF) and (4) designing a cloning strategy to assemble the final targeting DNA. SapTrap Builder provides an intuitive graphical interface that leads the user through these tasks. Importantly, the program handles the complicated and tedious elements of design and error checking, freeing the user to focus on the broader design.

To design reagents for a SapTrap assembly, the user pastes a genomic DNA sequence into the program’s main display text box. Once the genomic site is entered, the user is guided through the standard steps: translation frame selection, tag-type selection, insert site selection, guide RNA selection, repair template mutation, design review, and reagent sequence generation. For each step, the display is updated to show necessary sequence information, and modifications are made using simple keyboard inputs. The program highlights common design errors. By default, the program produces reagents for annealed oligo-based SapTrap assemblies. A user-modifiable options box allows changes to the output for synthetic gene fragment or PCR-based assemblies. Although our program is primarily designed for use with the SapTrap system, we included a ‘Generic’ option that produces only raw guide RNA and homology arm sequences, allowing users to utilize the guide RNA selector and mutator functionality with alternative DNA assembly strategies.

Directed genome editing is conceptually simple but practically complex. Our overall goal is to simplify design and production workflows to expand the use of genome editing in *C. elegans*. Together with our plasmid toolkit, SapTrap Builder greatly simplifies the design and assembly of complex targeting vectors for introducing both point mutations and genetic tags. Recently, the hygromycin-resistance self-excising cassette has been made compatible with SapTrap (Dickinson et al., 2018). In addition, we have generated new vectors to introduce point mutations into genes using *unc-119+* selection (Schwartz and Jorgensen, 2018). The SapTrap system accelerates the design and production of CRISPR reagents leaving only injection of constructs as the main rate-limiting step for the generation of edited worm strains.

## Reagents

The SapTrap Builder software, an instructional video, and an extensive PDF user guide can be downloaded at: http://jorgensen.biology.utah.edu/Labsite/resources.html

## References

[R1] Dickinson Daniel J., Slabodnick Mark M., Chen Alicia H., Goldstein Bob (2018). SapTrap assembly of repair templates for Cas9-triggered homologous recombination with a self-excising cassette. microPublication Biology.

[R2] Schwartz ML, Jorgensen EM (2016). SapTrap, a Toolkit for High-Throughput CRISPR/Cas9 Gene Modification in Caenorhabditis elegans.. Genetics.

[R3] Schwartz Matthew, Jorgensen Erik (2018). SapTrap vectors for introducing point mutations with *unc-119+* selection. microPublication Biology.

